# Miami Medical College

**Published:** 1865-10

**Authors:** 


### Miami Medical College.

The first term since the re-organization of this school, commences the first of November. It will be observed by reference to our advertisement sheet, that this school has fixed a *matricalation fee* of $15 00, and makes no charge for Professors' tickets.

